# *Wolbachia* detection in insects through LAMP: loop mediated isothermal amplification

**DOI:** 10.1186/1756-3305-7-228

**Published:** 2014-05-19

**Authors:** Daniela da Silva Gonçalves, Anna Paula Alvim Cassimiro, Caroline Dantas de Oliveira, Nilton Barnabé Rodrigues, Luciano Andrade Moreira

**Affiliations:** 1 Grupo: Mosquitos Vetores: Endossimbiontes e Interação Patógeno Vetor, Centro de Pesquisas René Rachou - Fundação Oswaldo Cruz, Av. Augusto de Lima 1715, 30190-002 Belo Horizonte, MG, Brazil; 2 Entomologia Médica, Centro de Pesquisas René Rachou - Fundação Oswaldo Cruz, Av. Augusto de Lima 1715, 30190-002 Belo Horizonte, MG, Brazil

**Keywords:** *Wolbachia*, LAMP-loop mediated isothermal amplification, Mosquito, Insect

## Abstract

**Background:**

The bacterium *Wolbachia* is a promising agent for the biological control of vector-borne diseases as some strains have the ability to block the transmission of key human disease-causing pathogens. Fast, accurate and inexpensive methods of differentiating between infected and uninfected insects will be of critical importance as field-based trials of *Wolbachia*-based bio-control become increasingly common.

**Findings:**

We have developed a specific and sensitive method of detecting *Wolbachia* based on the isothermal DNA amplification. This technique can be performed in an ordinary heat block without the need for gel-based visualisation, and is effective for a wide variety of insect hosts.

**Conclusion:**

Here we present the development of a rapid, highly sensitive and inexpensive method to detect *Wolbachia* in a variety of insect hosts, including key mosquito disease vectors.

## Findings

Vector-borne diseases (e.g. malaria, dengue, filariases and Chagas disease) occur in more than 100 countries, primarily within the tropics, with the annual, global death rate in the millions. Despite the existence of a variety of vector control measures disease incidence is generally increasing, and consequently there is an urgent need to develop new and effective control strategies [1]. Ideally these strategies should be able to be used in conjunction with existing control methods, and in this context the bacterium *Wolbachia pipientis* has proven to be a promising alternative, given its ability to restrict pathogen development in many different mosquito-pathogen combinations [2-5], and recently being applied in the field in Australia, Vietnam, Indonesia and soon in other dengue endemic countries (http://www.eliminatedengue.org).

*Wolbachia* is a vertically-transmitted bacterial endosymbiont of arthropods that is able to manipulate its host’s reproductive system and consequently spread rapidly through wild populations [6]. *Wolbachia* was originally identified in the ovaries of the mosquito *Culex pipiens*[7]*,* and recent studies have estimated that 40% of terrestrial arthropod species are infected with the bacterium [8]. Critically, infections do not occur in key vector species including the dengue mosquito *Aedes aegypti* or in the *Anopheles* genus [9]. *Wolbachia* is typically detected within the host by PCR against genes such as *wsp* (*Wolbachia* surface protein) [10], ftsZ (cell division protein) [11], and 16S ribosomal protein [12], or through fluorescence-based assays [13]. These techniques can be expensive to perform for large numbers of samples, and require the use of laboratory equipment.

The LAMP (loop mediated isothermal amplification) technique utilizes *Bst* DNA polymerase to create stand-displacement amplification, and operates at a constant temperature [14]. Examples of its use are very broad, ranging from the detection of pathogenic microorganisms to sex determination in embryos [15]. The LAMP product can be detected by the visualisation of turbidity, fluorescence or a metal ion indicator producing a colorimetric change [16] without the need to run a gel. These characteristics make LAMP a suitable method to detect *Wolbachia* under circumstances where minimum infrastructure is available. Here we report the use of LAMP to detect *Wolbachia* in a diverse range of insect species, which will prove beneficial to monitoring the progress of field-based trials utilising the bacterium.

## Methods

We examined many different species including laboratory-reared mosquito lines of *Aedes fluviatilis*, naturally infected by the *Wolbachia* strain *w*Flu*, A. aegypti* transinfected with the *w*MelPop and *w*Mel strains [17-19], and uninfected *Anopheles aquasalis* mosquitoes. These lines were reared at FIOCRUZ Minas as previously described [20]. We also examined field-captured mosquitoes including *A. aegypti, A. albopictus*, and *Culex sp.* Furthermore, insects belonging to a diverse range of orders, which were known to be *Wolbachia*-infected (unpublished results) were also included.

Specimens were stored in 96% ethanol until DNA extraction. Whole insect specimens were homogenized for 1.5 min using a Mini-Beadbeater-16 (BioSpec), in 1.5 mL tubes containing one 3 mm glass bead and 80 μl of homogenization buffer (10 mM Tris, 1 mM EDTA, 50 mM NaCl [pH 8.2]), and were then centrifuged at 4°C for 30s at 13,000 rpm [21]. The samples were then incubated at 97°C for 10 min, placed on ice for 15 min, and centrifuged for 10 min (4°C, 13,000 rpm). The supernatant was diluted 1:10. Primers for the *Wolbachia* 16S ribosomal sequence [12] were designed using the LAMP Designer 1.02 software (PREMIER Biosoft International) (Table 1).

**Table 1:** **Loop-mediated isothermal amplification (LAMP) primers based on *Wolbachia *** **16S sequences, designed using LAMP designer 1.02 software**

**Primer**	**Sequence (5′-3′)**
F3	CTCGTGTCGTGAGATGTTG
B3	GAACGTATTCACCGTGGC
FIP	CCCACTCCATAAGGGCCATGAGGGACTTTAAGGAAACTGCC
BIP	CAATGGTGGCTACAATGGGCTGGCAGAGTACAATCCGAACTG
LoopF	CCACCTTCCTCCAGTTTATCAC
LoopB	CGCGAGGCTAAGCTAATCC

Each LAMP reaction (V_total_ = 25 μl) contained 1× ThermoPol Reaction Buffer (20 mM Tris–HCl, pH 8.8, 10 mM KCl, 10 mM (NH_4_)_2_SO_4_, 2 mM MgSO_4_, 0.1% Triton X-100), 0.32 mM of each dNTP, 0.64 μM of each internal primer (FIP/BIP), 0.32 μM of LoopF and LoopB, 0.16 μM of each external primer (F3/B3), 0.64 M of Betaine, 6U of *Bst* DNA polymerase, large fragment (New England Biolabs), and approximately 30 ng of DNA. 1.2 mM of the metal ion indicator Hydroxy Naphthol Blue (HNB) was added to the ThermoPol Reaction Buffer. The mixture was incubated at 63°C for 90 minutes on a thermocycler or heat block, to determine if the reaction could be performed with a simpler setup. To check the sensitivity of the assay we cloned the external primer amplicon (F3/B3) into the pGemT-Easy (Promega) plasmid, and observed the efficacy of the assay on serial dilutions of the product.

## Results and discussion

Loop mediated isothermal amplification has been used to detect different organisms, such as *Leishmania*[22], *Toxoplasma gondii*[23] and also bacteria belonging to the same class as *Wolbachia* (Alphaproteobacteria); *Brucella* spp [24], *Anaplasma ovis*[25] and *Ehrlichia ruminantium*[26]*.* We designed a LAMP-based assay to detect *Wolbachia* in different hosts using the bacterial 16S rRNA sequence [12]. Initially we designed the four basic LAMP primers (F3, B3, FIP and BIP) but due to low specificity we then included the extra Loop primers [14].

To optimize the LAMP reaction for *Wolbachia,* samples of *A. fluviatilis* and *w*Mel-infected *A. aegypti* were used to conduct a time course assay where we determined that amplification first occurred after 60 minutes of incubation (at 63°C using a thermocycler – Additional file 1). This was subsequently standardized at 90 minutes in order to account for samples with low bacterial density.

**Figure 1:**
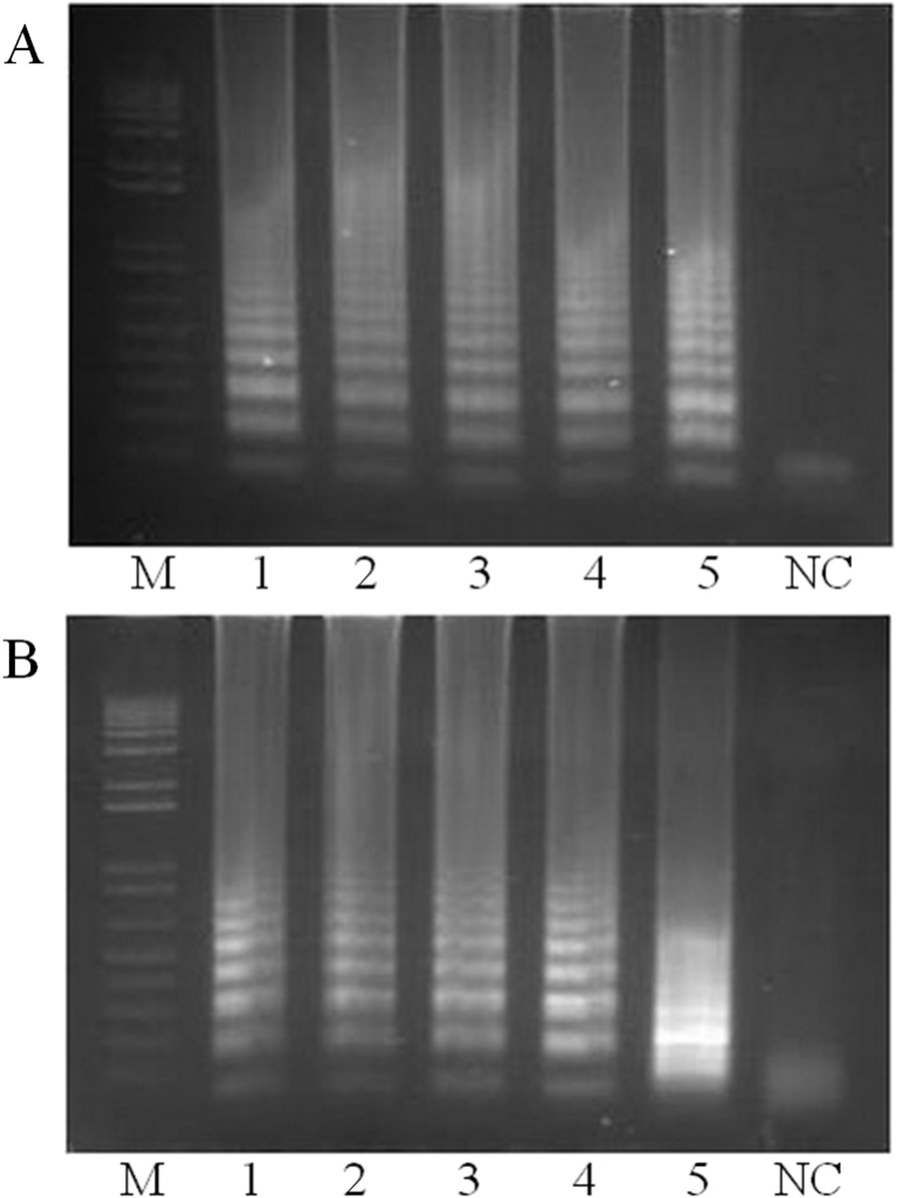
**Sensitivity of the LAMP assay.** To determine the sensitivity of our assay we cloned the product of the LAMP reaction into a plasmid and then performed the assay using serial dilutions at the following concentrations: 10^7^ (1), 10^5^ (2), 10^3^ (3), 10^1^ (4) and 10° (5). We performed the assay using both a thermocycler **(A)** and a heat block **(B)** with both proving suitable. Samples were incubated at 63°C for 90 min. The results were visualized on a 1.5% agarose gel stained with ethidium bromide. M = 1 Kb Plus, Invitrogen NC = negative control.

Assay sensitivity was assessed by serially diluting plasmid DNA containing the target sequence. Dilutions containing 10^7^, 10^5^, 10^3^, 10^1^ and 10° copies of the target gene were incubated for 90 min, using a thermocycler (Figure 1A) or heat block (Figure 1B). Sensitivity was high using either method, with amplification observed from as little as 10° copies of the target sequence, although it should be noted that amplification from plasmids is more effective than from genomic DNA.

**Figure 2:**
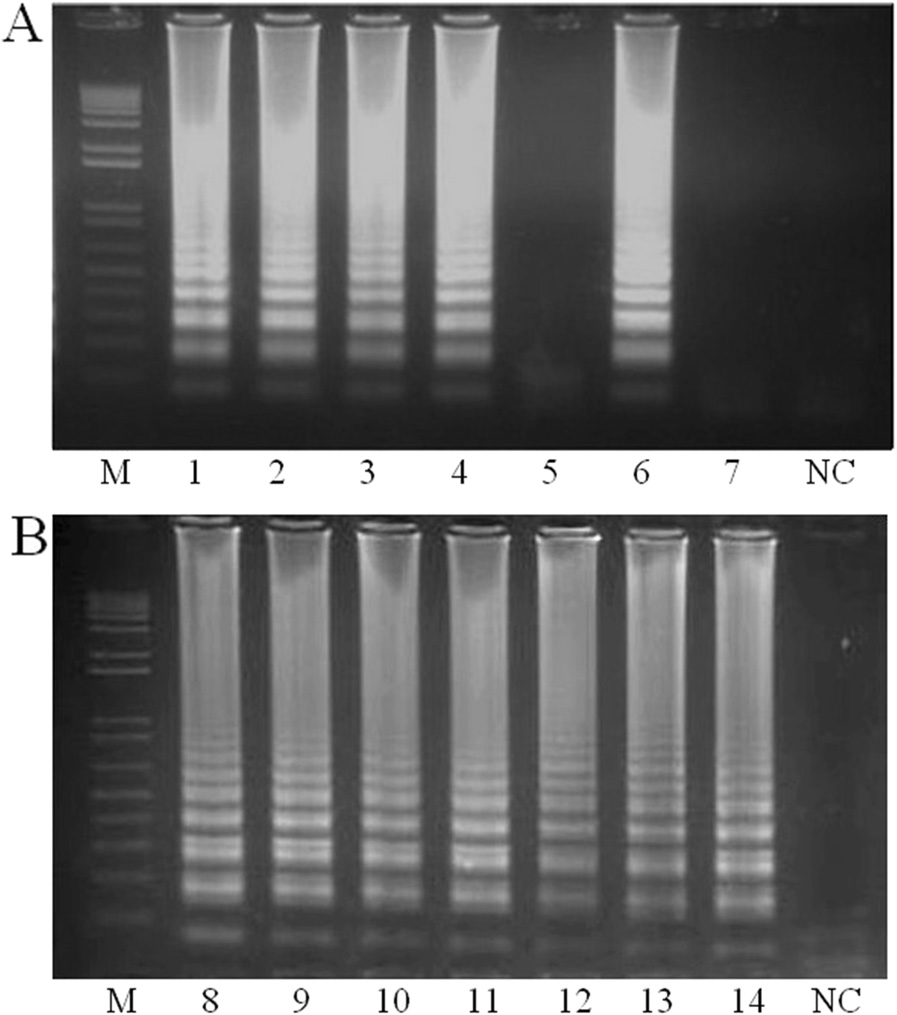
**Specificity and reliability of the LAMP assay in different insects.** To determine the specificity and reliability of our assay we amplified the DNA from different mosquito species (Diptera), including those naturally infected with *Wolbachia:***(A)***Ae. albopictus* (1), *Ae. fluviatilis* (2), *Culex sp.* (6) those with transinfections; *Ae. aegypti* – *w*Mel (3), *Ae. aegypti – w*MelPop (4), and those with no infection; *Ae. aegypti* (5), *An. aquasalis* (7)*.* We also examined insects from a range of different orders that were known to be infected with *Wolbachia:***(B)** Hymenoptera (8), Hemiptera (9), Lepidoptera (10), Orthoptera (11), Siphonaptera (12), Coleoptera (13), Isoptera (14). M = 1 Kb Plus, Invitrogen. NC = negative control. All *Wolbachia*-infected specimens amplified, while no amplification was observed for uninfected specimens indicating that the assay was both highly reliable and highly specific.

To check the specificity of our assay we analyzed mosquito specimens (Diptera: *Culicidae*) both infected and uninfected with *Wolbachia*. No amplification was observed in uninfected specimens, suggesting that the assay was highly specific and did not react to other bacteria infecting the mosquito gut [27]. We were also correctly able to identify *Wolbachia* infection in insect specimens from other orders (Figure 2B). It is important to mention though, as lateral gene transfer events are quite common in *Wolbachia*[28] further confirmation of the presence of this bacterium (MLST strain typing or in situ hybridization for instance) will likely be appropriate in specific studies, where the high throughput screening for positive individuals is not the main objective.

**Figure 3:**
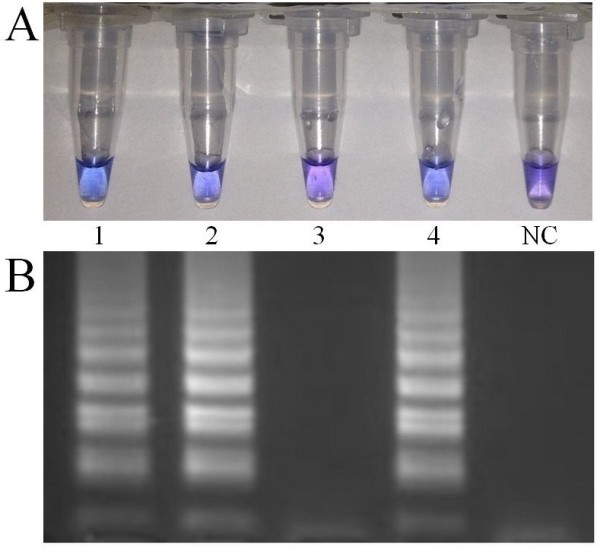
**Detection of *Wolbachia *infection by ion indicator visualization. (A) **With the addition of the ion indicator Hydroxy Naphtol Blue to the LAMP reaction it was possible to determine whether a mosquito specimen was *Wolbachia*-infected (blue), or -uninfected (purple) using the naked eye. The assay was highly specific with *Wolbachia-*infected *Ae. fluviatilis* (1), *Culex spp.* (2)*, Ae. aegypti* (4), all giving a positive result, while the uninfected *An. aquasalis* (3) did not. **(B)** Ethidium bromide stained gel with **(A)** samples NC = negative control.

The assay included the metal ion indicator Hydroxy Naphthol Blue [16] (which reacts with the pyrophosphate ion by-product of the reaction) to check whether we could detect *Wolbachia* without running agarose gels. We were able to clearly visualise a difference in colour after incubating the samples of *A. fluviatilis (*infected with *w*Flu) and *A. aegypti* (wild type and infected with *w*Mel), besides the negative *A. aquasalis,* for 150 min at 63°C in the thermocycler (Figure 3). This step could be utilized when access to laboratory equipment is limited and also has an advantage of reducing the risk of contamination (no need to open the tubes).

In terms of the cost per reaction, LAMP is approximately half as expensive as conventional PCR. If the cost of equipment is also included, LAMP becomes comparatively even cheaper given that it can be performed using only a heat block. As a further cost-saving measure, or if required in the field, homogenization of specimens could be performed using micro-pestles rather than a beadbeater.

We have demonstrated the reliability and sensitivity of using LAMP to detect *Wolbachia*, with detection possible for low-density infections, and the results easily visualized by the naked eye. Consequently, this technique could be readily applied in the field, without the need to use expensive laboratory equipment. This is the first report of the use of LAMP to detect *Wolbachia* infection in different hosts. The technique could potentially be applied to differentiating between multiple strains that could potentially occur within the same host species.

## Competing interests

The authors declare that they have no competing interests.

## Authors’ contributions

DSG participated in the design of the study, carried out all experiments and drafted the manuscript. APAC helped to conduct the experiments. CDO collected and screened infected insects. NBR and LAM participated on the study design. LAM coordinated the work, critically reviewed the manuscript and contributed to obtaining the results. All authors read and approved the final version of the manuscript.

## Supplementary Material

Additional file 1 **Standardization of the LAMP assay incubation time.** To determine the optimal incubation time for the assay, LAMP reactions were performed using; (1) *Ae. fluviatilis* naturally infected with *Wolbachia* (*w*Flu), and (2) *Ae. aegypti* artificially infected with *w*Mel. Samples were incubated at 63°C in a thermocycler and the tubes were removed at different times: 30 min (a), 60 min (b), 90 min (c), 120 min (d), 150 min (e). The products were visualised on a 1.5% agarose gel stained with EtBr. Visible products were present after 60 minutes of incubation. NC = negative control. Click here for file
