# Supplementary figures and images for: Wolbachia detection in insects through LAMP: loop mediated isothermal amplification
Source: Parasit Vectors. 2014 May 19;7:228. doi: 10.1186/1756-3305-7-228 (PMC4033683; doi:10.1186/1756-3305-7-228)

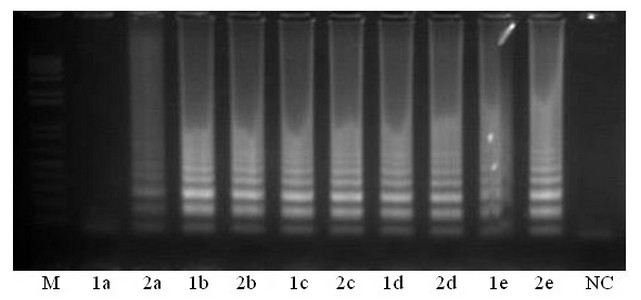

Supplement: Additional file 1 — Standardization of the LAMP assay incubation time. To determine the optimal incubation time for the assay, LAMP reactions were performed using; (1) Ae. fluviatilis naturally infected with Wolbachia (wFlu), and (2) Ae. aegypti artificially infected with wMel. Samples were incubated at 63°C in a thermocycler and the tubes were removed at different times: 30 min (a), 60 min (b), 90 min (c), 120 min (d), 150 min (e). The products were visualised on a 1.5% agarose gel stained with EtBr. Visible products were present after 60 minutes of incubation. NC = negative control. [file 1756-3305-7-228-S1.jpeg]
